# Immunogenicity and Safety of MF59-Adjuvanted Quadrivalent Influenza Vaccine Compared with a Nonadjuvanted, Quadrivalent Influenza Vaccine in Adults 50–64 Years of Age

**DOI:** 10.3390/vaccines11101528

**Published:** 2023-09-26

**Authors:** Airi Poder, Janine Oberije, Jay Meyer, Peter Heymer, Deborah Molrine, Eve Versage, Leah Isakov, Qiuhong Zhang, Matthew Hohenboken

**Affiliations:** 1 Tartu University Hospital, 50406 Tartu, Estonia; 2 CSL Seqirus, 1105 BJ Amsterdam, The Netherlands; 3 Velocity Clinical Research, Lincoln, NE 68510, USA; 4 Klinische Forschung Dresden GmbH, 01069 Dresden, Germany; 5 CSL Seqirus, Waltham, MA 02451, USA; 6 CSL Seqirus, Summit, NJ 07901, USA

**Keywords:** influenza, adjuvanted influenza vaccine, chronic medical condition, age 50–64 years, middle-aged adults

## Abstract

Adults aged 50–64 years have a high incidence of symptomatic influenza associated with substantial disease and economic burden each year. We conducted a randomized, controlled trial to compare the immunogenicity and safety of an adjuvanted quadrivalent inactivated influenza vaccine (aIIV4; n = 1027) with a nonadjuvanted standard dose IIV4 (n = 1017) in this population. Immunogenicity was evaluated on Days 22, 181, and 271. On Day 22, upper limits (UL) of 95% confidence intervals (CI) for geometric mean titer (GMT) ratios (IIV4/aIIV4) were <1.5 and 95% CI ULs for the difference in seroconversion rate (SCR IIV4 − aIIV4) were <10% for all four vaccine strains, meeting primary endpoint noninferiority criteria. Protocol-defined superiority criteria (95% CI ULs < 1.0) were also met for A(H1N1) and A(H3N2). Immune responses following aIIV4 vaccination were more pronounced in persons with medical comorbidities and those not recently vaccinated against influenza. Safety data were consistent with previous studies of MF59 adjuvanted seasonal and pandemic influenza vaccines. These findings support the immunological benefit of aIIV4 for persons aged 50–64 years, especially those with comorbidities.

## 1. Introduction

Seasonal influenza is associated with substantial disease and economic burden each year, and adults aged 50–64 years have the highest incidence of symptomatic influenza in the adult population. In the United States (US), the median incidence of influenza between 2010 and 2016 among those aged 50–64 years was 12.0% compared with 7.4% in persons aged 18–49 years and 3.9% in persons aged ≥65 years [1]. During the 2018–2019 influenza season (prior to the COVID-19 pandemic), the estimated rate of hospitalizations due to influenza disease in the US was three times higher in adults aged 50–64 years than in those aged 18–49 years (121.3 vs. 39.8 per 100,000), and outpatient visit rates were more than twice as high compared with those of older adults aged ≥65 years (4918.90 vs. 2400.60 per 100,000 corresponding to 3.11 million vs. 1.26 million visits) [2]. A recent systemic literature review of the economic burden of seasonal influenza found that both direct costs of hospitalization and indirect costs of absenteeism from work or as caretaker and lost productivity due to death were higher in adults aged 50–64 years than those aged 18 to 49 years [3].

The use of seasonal influenza vaccines is recommended to provide protection against influenza illness and potential complications, most commonly hospitalization for pneumonia. Annual influenza vaccination is recommended in the US for all persons 6 months of age and older, particularly for those at high risk of severe influenza-associated complications, such as all adults aged ≥50 years as well as persons with comorbidities, including cardiovascular disease (CVD), obesity and diabetes, chronic kidney disease (CKD), and chronic pulmonary diseases, and those who are immunocompromised [4]. In Europe, influenza vaccination is recommended for persons with chronic medical conditions at risk of severe disease, and some countries in Europe and Asia recommend vaccination for all adults from ages ≥50, ≥55, or ≥60 years [5,6,7,8]. Despite these recommendations, vaccine uptake in persons between 50 and 64 years is lower than among those aged ≥65 years [9,10,11].

Strategies to enhance seasonal influenza vaccine effectiveness, particularly for older adults, include formulating vaccines with higher vaccine antigen content or use of an adjuvant [4,12,13]. MF59-adjuvanted seasonal influenza vaccines have been approved for use in adults 65 years of age and older since 1997. The MF59^®^ adjuvant is a squalene-based, oil-in-water emulsion shown to induce robust anti-HA titers to homologous and heterologous strains in humans [14,15,16,17,18] and to protect against lethal challenge to heterologous influenza in mouse models [19,20]. The adjuvant effect of MF59 to enhance antibody and T-cell responses has been extensively studied, demonstrating promotion of T-cell activation and B-cell expansion as well as other T-cell-mediated immune responses [21,22,23]. In clinical studies, compared to non-adjuvanted vaccines, MF59-adjvanted trivalent influenza vaccines have been shown to increase the magnitude and breadth of the immune response in adults ≥65 years as well as those younger than 65 years who have chronic medical conditions that increase their risk of influenza-associated complications [14,24,25,26]. Further evaluation of the potential benefit of MF59-adjuvanted seasonal influenza vaccine is warranted in all persons younger than 65 years of age.

In the present study, we compared the immunogenicity and safety of an MF59-adjuvanted quadrivalent inactivated influenza vaccine (aIIV4) with that of a licensed, standard-dose quadrivalent inactivated influenza vaccine (IIV4) in a population aged 50–64 years during the 2021–2022 influenza season.

## 2. Materials and Methods

### 2.1. Study Design

This phase 3, randomized, comparator-controlled, observer-blind, multicenter study evaluated the immunogenicity and safety of aIIV4 compared with IIV4 in subjects 50 to ≤64 years of age. The study was conducted at 29 centers in Estonia, Germany, and the US between 30 September 2021 and 9 September 2022. This study was designed, implemented, and reported in accordance with ICH Harmonised Tripartite Guidelines for Good Clinical Practice [27], the ethical principles laid down in the Declaration of Helsinki [28], and applicable local laws and regulations. The study protocol and consent form were approved by each site’s institutional review board or ethics committee. All participating subjects provided written, informed consent.

### 2.2. Study Population

Eligible male and female subjects were 50 to ≤64 years of age in general good health and/or with stable comorbidities. Female subjects with child-bearing potential were required to be using effective birth control for at least 30 days before providing informed consent and 2 months after receiving the vaccine. The main exclusion criteria included acute (severe) febrile illness; any medical conditions prespecified as adverse events of special interest (AESI); receipt of any influenza vaccine within 6 months prior to enrollment; hypersensitivity, including allergy, to any of the vaccine components; abnormal function of the immune system from a clinical condition or use of immunosuppressive or immunomodulating agents and clinical conditions contraindicating intramuscular vaccination and blood draws. At screening, the site investigator completed an assessment of factors that are part of a validated prediction rule for estimating the probability of hospitalization due to pneumonia or influenza and death from any cause in elderly persons [29]. The applicable characteristics used to calculate a prognostic score include age, male sex, outpatient visits during previous year, previous hospitalizations due to pneumonia or influenza, and history of hematological or non-hematological cancer; dementia or stroke; pulmonary disease; renal disease or transplant; or heart disease. Using this prediction rule, a score of <50 is considered lower risk and a score of ≥50 is considered higher risk for serious complications from influenza [29].

An interactive response technology system was used to randomly assign subjects to receive either aIIV4 or IIV4 in a 1:1 ratio, stratified by age (50 to ≤59 years and 60 to ≤64 years) and whether or not the subject had received any influenza vaccination within the previous 3 influenza seasons.

### 2.3. Study Vaccines and Procedures

Vaccines were administered as a single intramuscular injection on Day 1, preferably in the deltoid muscle of the nondominant arm. Both aIIV4 (Fluad^®^ Quadrivalent, Seqirus UK Limited, Maidenhead, UK) and IIV4 (Fluarix Tetra, GSK, Brentford, UK) consisted of a 0.5 mL dose containing 15 μg of hemagglutinin (HA; total of 60 μg in the vaccine) of each strain as recommended by WHO for the Northern Hemisphere 2021–2022 season. Both aIIV4 and IIV4 included A(H1N1) strain A/Victoria/2570/2019 (IVR-215) and B/Yamagata strain B/Phuket/3073/2013 (BVR-1B). In IIV4, the A(H3N2) strain was A/Tasmania/503/2020 (IVR-221), an A/Cambodia/e0826360/2020 (H3N2)-like virus, and the B/Victoria strain was B/Washington/02/2019; aIIV4 included A(H3N2) strain A/Cambodia/e0826360/2020 (IVR-224) and B/Victoria strain B/Victoria/705/2018 (BVR-11), a B/Washington/02/2019-like virus. In addition, aIIV4 contained 9.8 mg of MF59 adjuvant per 0.5 mL dose.

The study comprised a treatment period extending from the time of vaccination to 21 days after vaccination (i.e., Day 1 to Day 22) and a follow-up period, extending from Day 23 to the study’s end on Day 271. During the treatment period, safety was assessed via collection of solicited adverse events (AE) and body temperature from day 1 through day 7 after vaccination (or longer if the events were not resolved), as recorded by the subject using an eDiary, and reports of all unsolicited adverse events during a safety phone call on Day 15 and at the Day 22 clinic visit. In the follow-up period, reports of serious adverse events (SAE), AESIs, and AEs leading to study withdrawal were collected via spontaneous reporting and at scheduled visits (phone call on Day 91; clinic visits on Days 181 and 271). Immunogenicity was assessed through validated hemagglutination inhibition (HI) assays performed on serum samples collected before vaccination on Day 1 and on Days 22, 181, and 271 via titration of homologous anti-HA antibodies against each vaccine strain, using egg-derived target viruses (Viroclinics Bioscience B.V., Rotterdam, The Netherlands).

### 2.4. Data Sets

The all-enrolled set included all screened subjects who provided informed consent and provided demographic and/or baseline screening assessments, whereas the all-exposed set included all enrolled subjects who received a study vaccination. The immunogenicity full analysis set (FAS) included all enrolled subjects who were randomized, received study vaccination, and provided immunogenicity data at any time point. The immunogenicity per-protocol set (PPS) included all FAS subjects who had both a Day 1 and Day 22 immunogenicity assessment, received the vaccine to which the subject was randomized, had no protocol deviations leading to exclusion, and were not excluded for other reasons defined prior to unblinding for analysis. The solicited safety set included all exposed subjects with any solicited AE data, including body temperature measurements or use of analgesics/antipyretics, and the unsolicited safety set included all exposed subjects who provided unsolicited AE data. The overall safety set included all subjects in the solicited safety set and/or unsolicited safety set.

### 2.5. Study Endpoints

#### 2.5.1. Primary Immunogenicity Endpoints

Immunogenicity was assessed for each vaccine group via evaluation of pre- and postvaccination geometric mean titers (GMT) and seroconversion rate (SCR). SCR was defined as the percentage of subjects with either a prevaccination HI titer < 1:10 and a postvaccination HI titer ≥ 1:40 or a prevaccination HI titer ≥ 1:10 and a ≥4-fold increase in postvaccination titer. Noninferiority of aIIV4 vs. IIV4 for each homologous egg-derived vaccine strain—A(H1N1), A(H3N2), B/Victoria, and B/Yamagata—was evaluated at Day 22 in the immunogenicity PPS population based on GMT ratios (GMT IIV4/GMT aIIV4) and the difference in seroconversion rate (SCR IIV4 − SCR aIIV4). The study was considered a success if the following noninferiority criteria were met: (1) the upper limit of the 95% confidence interval (CI) was ≤1.5 for the GMT ratio and (2) the upper limit of the 95% CI was ≤10% for the SCR difference for each of the four vaccine strains.

The superiority of the immune response was evaluated using the GMT ratio (IIV4/aIIV4) in the immunogenicity FAS population. The protocol-specified superiority criterion was met if the upper limit of the 95% CI was <1.0 for at least two vaccine strains.

#### 2.5.2. Secondary Immunogenicity Endpoints

Secondary immunogenicity endpoints included persistence of the immune response based on the GMT ratio (IIV4/aIIV4) at Day 181; HI GMTs on Days 1, 22, and 181; geometric mean fold increase (GMFI), defined as the geometric mean of postvaccination HI titer on Day 22 or Day 181 divided by the Day 1 prevaccination HI titer; the percentage of subjects with a HI titer ≥ 1:40 at Days 1, 22, and 181; and SCR on Days 22 and 181.

An exploratory analysis evaluated persistence of the immune response at Day 271 based on GMTs, GMT ratios, and GMFI for all strains. For this analysis, Day 1 serum samples were retested at the time of the Day 271 testing campaign given the inherent variability in the HI assay.

#### 2.5.3. Safety Endpoints

Reactogenicity and safety were assessed based on the frequency and severity of solicited local and systemic AEs on Day 1 through Day 7; all unsolicited AEs reported on Day 1 through Day 22; and SAEs, AEs leading to withdrawal from the study, and AESIs reported on Day 1 through Day 271.

### 2.6. Statistical Analyses

The analysis model for the HI GMT used a general linear model on log-transformed (base 10) titers from Days 22, 181, and 271 as the outcome variable and used vaccine group, age cohort, sex, history of any influenza vaccination within the previous 3 influenza seasons, and prevaccination titer (log10 transformed) as covariates; from this model, adjusted differences in the least-square means (on the log10 scale) were produced with 95% CI for IIV4 vs. aIIV4. The estimated difference and the confidence limits were back-transformed to obtain adjusted GMT ratios with 95% CI. Assessment of the HI primary endpoint was based on the adjusted GMT ratio, and each of the four strains was analyzed separately. For descriptive presentation of the immune response, unadjusted estimates for GMTs, GMFIs, and pertaining two-sided 95% CIs were calculated assuming a log-normal distribution of the titers and completed by providing minimum, maximum, and median titers for each vaccine group. Binary data (i.e., percentages of subjects with seroconversion) were summarized for each vaccine strain within each vaccine group using crude estimates and reported together with two-sided 95% CIs calculated according to the Clopper and Pearson method; 95% CIs for the SCR differences were calculated using the Miettinen and Nurminen method [30,31]. All statistical analyses were performed using SAS Software, Version 9.4 or higher.

Hypothesis testing was performed sequentially according to the order of the objectives to keep the Family Wise Error Rate (FWER) at 0.05. Statistical testing proceeded in the following order at two-sided alpha 0.05: Day 22 noninferiority assessed by GMT ratio and SCR difference for all four strains, followed by Day 22 superiority assessed based on the GMT ratio for at least 2 of 4 strains. Pre-specified subgroup analyses by age cohort (50–59 and 60–64 years), previous vaccination within 3 years (yes or no), and comorbidity score (<50 or ≥50) were evaluated descriptively.

Safety data are presented descriptively, with percentages of subjects reporting any AE presented by vaccine group. Solicited AEs were summarized according to defined severity grading scales. Unsolicited AEs are summarized and presented by the Medical Dictionary for Regulatory Activities (MedDRA) preferred term classification (MedDRA version 25.0).

## 3. Results

### 3.1. Study Population

A total of 2044 subjects were enrolled and randomized, of which 2043 were exposed to the study vaccine (1 subject in IIV4 group did not receive the study vaccine). The immunogenicity FAS included 1027 subjects assigned to aIIV4 and 1016 assigned to IIV4. The PPS included 983 aIIV4 recipients and 985 IIV4 recipients. The solicited safety set included 1020 and 1008 subjects from the aIIV4 and IIV4 groups, respectively (Supplementary Figure S1), and the unsolicited safety set and overall safety set included 1027 subjects exposed to aIIV4 and 1016 subjects exposed to IIV4.

Baseline characteristics were similar between vaccine groups (Table 1). The majority (61.2%) of the subjects were female; 95.6% were white, and 58% had received at least one influenza vaccine in the preceding three influenza seasons. The mean BMI in the study population was 30.2 ± 6.7 kg/m^2^. Most subjects were healthy, with a comorbidity score <50.

### 3.2. Immunogenicity Data

**Figure 1:**
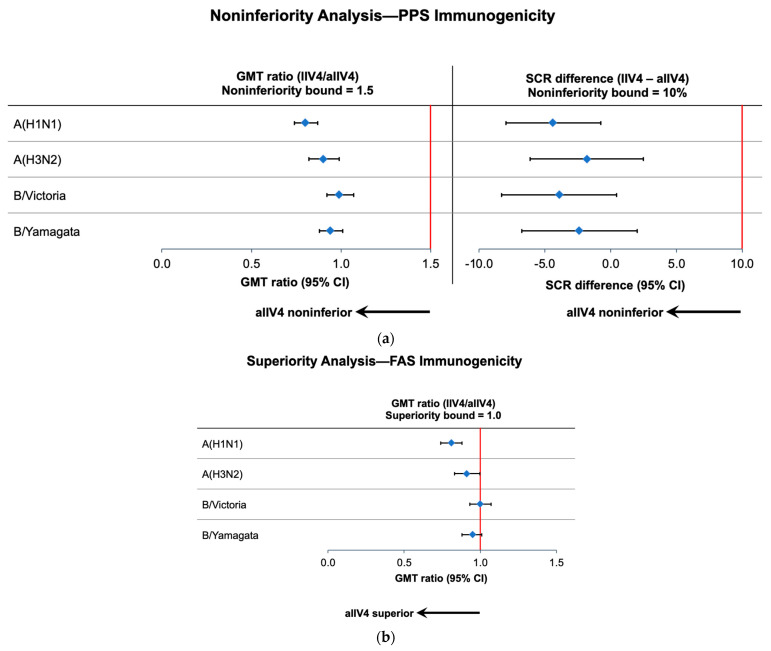
GMT ratios and SCR differences on Day 22. Blue diamonds represent the point estimates and cross-hatched lines the 95% CIs. (**a**) Noninferiority analysis in the PPS immunogenicity population. Noninferiority bounds are shown by solid red lines. (**b**) Superiority analysis in the FAS immunogenicity population. Superiority bound shown by solid red line. Abbreviations: aIIV4—adjuvanted quadrivalent inactivated influenza vaccine; CI—confidence interval; FAS—full analysis set; GMT—geometric mean ratio; IIV4—nonadjuvanted quadrivalent inactivated influenza vaccine; PPS—per protocol set; SCR—seroconversion rate.

The derived GMT ratios and SCR differences demonstrated that aIIV4 was noninferior to IIV4 for all vaccine strains (Figure 1a; Supplementary Table S1).

GMT ratios of IIV4/aIIV4 on Day 22 showed that aIIV4 was superior to IIV4 for A(H1N1) and A(H3N2) based on the FAS (Figure 1b; Supplementary Table S2), with a GMT ratio of 0.808 (95% CI, 0.745–0.876) for A(H1N1) and 0.910 (95% CI, 0.829–0.998) for A(H3N2). The upper limits of the 95% CIs were >1 for the B-lineage strains. In a sensitivity analysis based on the PPS, the superiority of aIIV4 was also observed for both A subtype vaccine strains (Supplementary Table S2). Unadjusted analysis data for secondary immunogenicity endpoints at all time points for each vaccine group can be found in Supplementary Table S3.

#### 3.2.1. Subgroup Analyses

**Figure 2:**
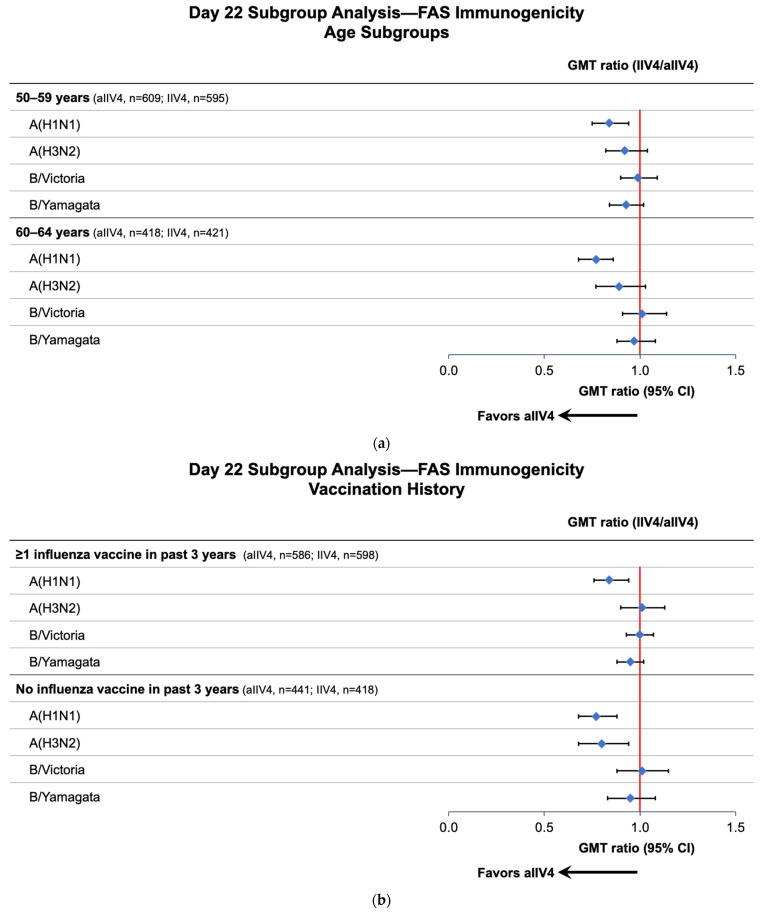

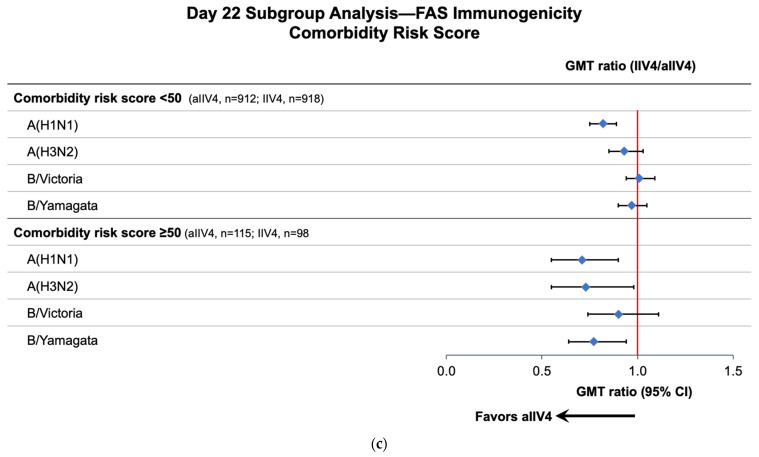
Day 22 GMT ratios (IIV4/aIIV4) by age (**a**), vaccination history (**b**), and comorbidity risk (**c**) subgroups (FAS Immunogenicity population). Abbreviations: aIIV4—adjuvanted quadrivalent inactivated influenza vaccine; CI—confidence interval; FAS—full analysis set; GMT—geometric mean ratio; IIV4—nonadjuvanted quadrivalent inactivated influenza vaccine. A GMT ratio <1 favors aIIV4.

For the analysis of age subgroups, the pattern of immune responses was similar to that in the overall population (Figure 2a). Day 22 GMT was notably higher for the A(H1N1) strain of the aIIV4 group compared to that of the IIV4 group for both age cohorts. Point estimates for the A(H3N2) strain GMT ratio for both age subgroups were similar to those observed in the overall study population; however, the 95% CIs were wider due to the smaller sample sizes and crossed the value of one. No notable differences between vaccine groups were observed in the B strain analyses for either age subgroup.

For subjects who had not been vaccinated within the past 3 years, Day 22 immune responses against A(H1N1) and A(H3N2) were notably higher in the aIIV4 than the IIV4 group; responses to B strains were not substantially different (Figure 2b). Among subjects who had received at least one influenza vaccination in the past 3 years, A(H1N1) immune response was notably higher in the aIIV4 group.

In subjects with a comorbidity score ≥50, suggesting a higher risk of hospitalization due to influenza-associated complications, Day 22 immune responses against A(H1N1), A(H3N2), and B/Yamagata were notably higher in the aIIV4 than the IIV4 group; the response to B/Victoria was not substantially different between the vaccine groups (Figure 2c). Among subjects with a comorbidity score <50, the pattern of results was similar to that in the overall population, with a higher immune response observed for the A(H1N1) strain in the aIIV4 group.

#### 3.2.2. Persistence of Response at 6 and 9 Months

At 6 and 9 months after vaccination, GMTs remained higher than the baseline, particularly for the A virus subtypes. The GMFIs for A(H1N1) in aIIV4 group were 13.07. 6.22 and 5.63 at Day 22, 181 and 271, respectively, and were 11.00, 5.47 and 4.94, respectively for the IIV4 group. The GMFIs for A(H3N2) at Day 22, 181, and 271 in the aIIV4 group were 7.09, 3.29 and 2.67, respectively; and 6.31, 3.08 and 2.44, respectively, in the IIV4 group. For both vaccine groups, the GMFIs for B/Victoria and B/Yamagata were approximately 3.5-fold on Day 22, 2-fold on Day 181 and 1.6-fold on Day 271. At 6 months (Day 181) after vaccination, GMT values were higher in the aIIV4 than the IIV4 group for A(H1N1), with a GMT ratio of 0.870 (95% CI, 0.803–0.944). The same pattern was observed at 9 months (Day 271) for A(H1N1), with a GMT ratio of 0.892 (95% CI, 0.815–0.976). There were no notable differences for the other vaccine strains at Day 181 and Day 271 (Supplementary Table S4).

### 3.3. Safety

**Figure 3:**
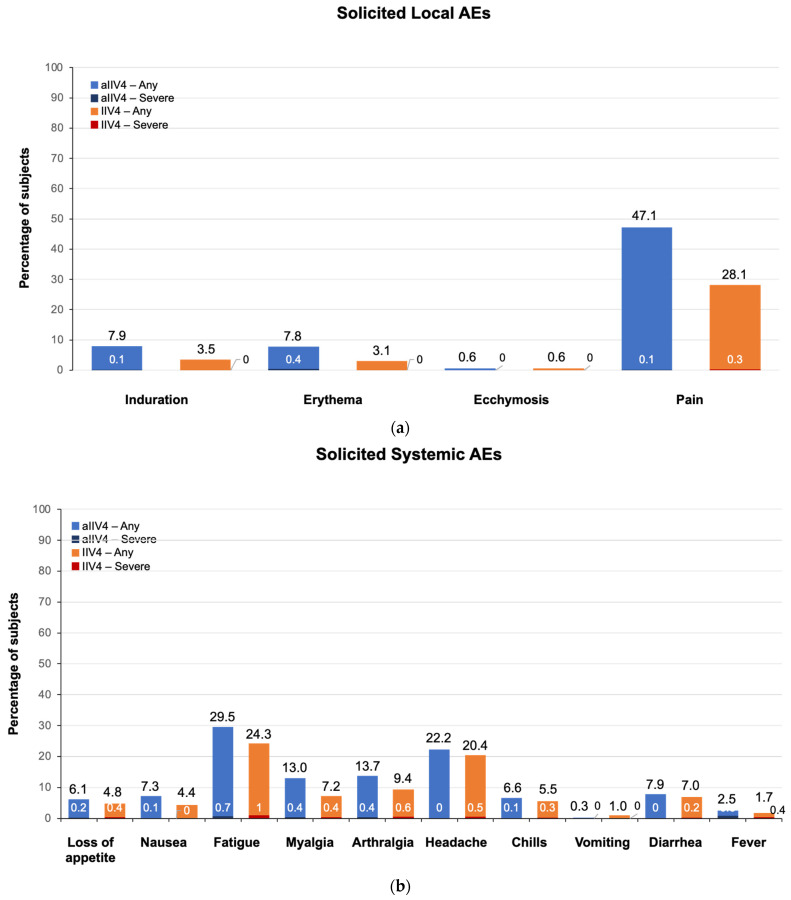
Percentage of subjects reporting solicited local (**a**) and systemic (**b**) adverse events (AE). Numbers at the top of the bars represent percentage of subjects reporting event of any severity grade; numbers within or at the bottom of the bars represent percentage reporting event as severe.

In the 7-day period after vaccination, the percentage of subjects reporting any solicited local and/or systemic AE was 65.9% in the aIIV4 group and 53.7% in the IIV4 group. Any solicited local AE was reported by 49.8% of aIIV4 recipients and 30.4% of IIV4 recipients. The most frequently reported solicited local AE was injection site pain, which was reported by 47.1% of the aIIV4 group and 28.1% of the IIV4 group (Figure 3a). The majority of solicited AEs were of short duration and rated as mild; less than 0.5% of subjects reported severe local AEs (Figure 3a).

Solicited systemic AEs were reported by 45.3% and 40.0% of the aIIV4 and IIV4 groups, respectively. The most frequently reported solicited systemic AEs were fatigue (aIIV4, 29.5%; IIV4, 24.3%) and headache (aIIV4, 22.2%; IIV4, 20.4%) (Figure 3b). Most were mild and of short duration; ≤1.0% of subjects in either vaccine group reported any single systemic AE as severe. In total, 35 of 1020 subjects (3.4%) in the aIIV4 group and 40 of 1008 subjects (4.0%) in the IIV4 group reported solicited systemic AEs that remained ongoing after Day 7. Use of antipyretic/analgesics for treatment or prevention of pain or fever was similar between the aIIV4 and IIV4 groups (12.9% vs. 9.6%).

Overall, the incidence of unsolicited AEs, including related AEs and SAEs, was similar between vaccine groups over the study period (Table 2). There was one death in the aIIV4 group due to lung adenocarcinoma, which was assessed as unrelated to the study vaccine. Two AESIs were reported in the aIIV4 group: one subject reported worsening of rheumatoid arthritis on study Day 164 and another subject reported autoimmune thyroiditis on study Day 228. Both events were assessed as unrelated to the study vaccine. One related SAE in the IIV4 group (hypertensive crisis on Day 1) occurred in a subject with multiple cardiometabolic comorbidities. The most common unsolicited AEs between Day 1 and 22 were nasopharyngitis, rhinitis, and headache.

## 4. Discussion

This is the first randomized controlled study to evaluate the immunogenicity and safety of aIIV4 in a population aged 50–64 years, an age group that contributes significantly to the burden of influenza disease both from a medical and economic perspective. The study showed the adjuvanted vaccine is safe and well tolerated and able to produce a strong immune response. Functional antibody responses with aIIV4 were notably higher than nonadjuvanted comparator vaccine for both A strains 3 weeks after vaccination, and the higher response for A(H1N1) persisted for 6 and 9 months, suggesting a durable effect of the adjuvanted vaccine.

For adults over the age of 50 years, the ability to respond optimally to vaccination is affected by immunosenescence, in which advancing age diminishes the effectiveness of the immune system [32]. Defects observed include impaired function of antigen-presenting cells, decreased capacity of T cells to respond to new antigens, reduced magnitude of antibody responses, decreased production of high-affinity antibodies, and diminished metabolic activity within memory CD4+ cells [32,33,34,35]. Immune responses against conventional trivalent influenza vaccines in adults ≥58 years of age have been shown to be 10%–23% lower than in adults younger than 58 years of age [36]. Enhanced influenza vaccines such as aIIV4 have been approved for adults aged 65 years and older, and with variability in the age of onset of immunosenescence, the immunological responses in this study suggest a potential role of adjuvanted influenza vaccine in individuals 50–64 years of age, particularly among those with comorbidities.

For the subgroup analyses comparing the primary Day 22 immune responses of aIIV4 to IIV4, higher GMT responses to multiple vaccine strains were observed for subjects with a high comorbidity score and for persons who had not been immunized for at least 3 years with seasonal influenza vaccine. These findings are consistent with results from previous studies involving persons aged <65 years comparing MF59-adjuvanted influenza vaccines to nonadjuvanted IIV that showed enhanced immune responses with adjuvanted vaccine in persons with chronic medical conditions [24,25]. In a randomized trial comparing adjuvanted with nonadjuvanted trivalent vaccine in patients 19–64 years with CKD undergoing dialysis, significantly higher seroconversion rates and GMT ratios against all three strains were observed with adjuvanted vaccine [24]. Two other randomized studies of subjects aged 18–60 years with underlying comorbidities (CVD; lung, liver or renal insufficiency; and/or diabetes) showed that aIIV3-vaccinated subjects had significantly higher HI titers against A(H3N2) and B [25] or against all three vaccine strains [37] than subjects who received IIV3 nonadjuvanted vaccine.

The prevalence of chronic conditions increases with age, and many underlying conditions are associated with an increased risk of influenza-related complications [38,39]. In a 2018 report from the National Foundation of Infectious Diseases, nearly 50% of US adults aged 45–64 years had two or more chronic health conditions [40]. For the US 2017–2018 influenza season, which was noted as a high-severity season, the highest influenza-associated hospitalization rates were observed among those 50 years and older; in this group, the most commonly reported underlying medical conditions were CVD; metabolic disorders such as diabetes and obesity; and chronic lung disease [41].

The finding that adjuvanted influenza vaccines may be particularly effective in adults aged <65 years with chronic medical conditions has broad implications for public health. In many countries, individuals with comorbidities are considered a high-priority group for influenza vaccination [4,5,6,7,8]. A recent study showed that the risk of influenza-related hospitalization increases as the number of influenza risk factors increases. Notably, persons aged 50–64 years with three or more comorbidities may be at even greater risk of influenza hospitalization than those aged ≥65 years with the same number of comorbidities [42]. Costs associated with influenza hospitalizations are up to 2.5 times higher in patients with comorbidities vs. those without them [3]. Indirect costs due to absenteeism and lost productivity due to death account for 83–99% of the total burden of influenza, and these indirect costs are higher in persons aged 50–64 than 18–49 years [3,43,44]. Like hospitalization costs, influenza absenteeism costs are higher in persons with chronic medical conditions than in those without them [45].

Influenza vaccine coverage is lower among those aged 50–64 years than those ≥65 years. We observed higher responses to both A subtype vaccine strain for subjects who had not received an influenza vaccine in the previous three influenza seasons in the aIIV4 group. Annual vaccination, however, is recommended for optimal protection from influenza disease due to changes in influenza strains circulating in a given season. Findings in this study also showed a benefit of an adjuvanted vaccine for those recently vaccinated.

Overall, aIIV4 was well tolerated with an acceptable reactogenicity and safety profile for the population aged 50–64 years. A small increase in subjects reporting local injection site pain with aIIV4 has been observed in adults older than 64 years and was likewise mainly mild and of short duration [46,47]. Solicited local and systemic adverse reactions were consistent with the well-established safety profile of MF59-adjuvanted pandemic influenza vaccines for this age group.

Limitations of the study include the duration of the study, which was conducted over only a single influenza season. Although both vaccines contain a nominal 15 µg of HA from each of the four vaccine strains, the vaccines are produced on two different manufacturing platforms; the split virion QIV contains more influenza proteins than aQIV, which is purified to contain only HA and neuraminidase antigens. Strengths of this randomized, controlled trial included a large sample size to assess safety and immunogenicity outcomes. In addition, the durability of immune responses was evaluated up to 9 months after vaccination.

## 5. Conclusions

The MF59-adjuvanted influenza vaccine demonstrated a positive benefit–risk profile for prophylaxis against influenza among individuals 50–64 years of age, with noninferior immunogenicity to all strains and higher responses to the A(H1N1) and A(H3N2) strains when compared to a licensed nonadjuvanted influenza vaccine in this randomized trial. An age-extension of the licensure of aIIV4 could further reduce the medical and economic burden of influenza on society.

## Figures and Tables

**Table 1:** Baseline characteristics of all enrolled subjects.

Characteristic		aIIV4 (n = 1027)	IIV4 (n = 1017)	Total (N = 2044)
Age (years), mean ± SD		57.8 ± 4.2	57.8 ± 4.2	57.8 ± 4.2
Age group, n (%)	50–59 years	609 (59.3)	596 (58.6)	1205 (59.0)
	60–64 years	418 (40.7)	421 (41.4)	839 (41.0)
Female sex, n (%)		635 (61.8)	615 (60.5)	1250 (61.2)
Race and ethnicity, n (%)	American Indian or Alaskan Native	2 (0.2)	3 (0.3)	5 (0.2)
	Asian	2 (0.2)	4 (0.4)	6 (0.3)
	Black	39 (3.8)	36 (3.5)	75 (3.7)
	Native Hawaiian or Pacific Islander	1 (0.1)	1 (0.1)	2 (0.1)
	White	982 (95.6)	972 (95.6)	1954 (95.6)
	Other	1 (0.1)	1 (0.1)	2 (0.1)
	Hispanic or Latino ethnicity	14 (1.4)	12 (1.2)	26 (1.3)
Country, n (%)	Estonia	391 (38.1)	396 (38.9)	787 (38.5)
	Germany	259 (25.2)	254 (25.0)	513 (25.1)
	United States	377 (36.7)	367 (36.1)	744 (36.4)
Influenza vaccination, previous 3 seasons, n (%)	Yes	586 (57.1)	598 (58.8)	1184 (57.9)
	No	441 (42.9)	419 (41.2)	860 (42.1)
Comorbidity, n (%)	Dementia or stroke	19 (1.9)	17 (1.7)	36 (1.8)
	Heart disease	95 (9.3)	113 (11.1)	208 (10.2)
	Non-hematological and hematological cancer	46 (4.5)	42(4.1)	88 (4.3)
	Pulmonary disease	115 (11.2)	92 (9.0)	207 (10.1)
	Renal disease or renal transplant	6 (0.6)	11 (1.1)	17 (0.8)
Comorbidity risk score ^a^, n (%)	<50	912 (88.8)	919 (90.4)	1831 (89.6)
	≥50	115 (11.2)	98 (9.6)	213 (10.4)
BMI (kg/m^2^), mean ± SD		30.1 ± 6.6	30.3 ± 6.8	30.2 ± 6.7

Abbreviations: aIIV4, adjuvanted quadrivalent inactivated influenza vaccine; BMI, body mass index; IIV4, nonadjuvanted quadrivalent inactivated influenza vaccine; SD, standard deviation. ^a^ Score of <50 is considered lower risk and a score of ≥50 is considered higher risk of serious influenza complications based on age, male sex, outpatient visits during previous year, previous hospitalizations due to pneumonia or influenza, and history of hematological or non-hematological cancer; dementia or stroke; pulmonary disease; renal disease or transplant; heart disease [29].

**Table 2:** Summary of unsolicited adverse events.

Event, n (%)		aIIV4 (n = 1027)	IIV4 (n = 1016)
Day 1–22	Any AE	169 (16.5)	172 (16.9)
	Any related AE	33 (3.2)	32 (3.1)
	Any severe AE	2 (0.2)	7 (0.7)
Day 1–271	SAE	31 (3.0)	31 (3.1)
	Related SAE	0	1 (0.1)
	AE leading to study withdrawal	0	1 (0.1)
	AESI	2 (0.2)	0
	Death ^a^	1 (0.1)	0
AEs occurring in >1% of subjects (preferred term), Days 1–22	Nasopharyngitis	16 (1.6)	10 (1.0)
	Rhinitis	15 (1.5)	11 (1.1)
	Headache	10 (1.0)	13 (1.3)

^a^ Lung adenocarcinoma, assessed as not related to study vaccine.

## Data Availability

Data available from the authors upon request.

## Supplementary Materials

The following supporting information can be downloaded at: https://www.mdpi.com/article/10.3390/vaccines11101528/s1, Figure S1. Subject disposition; Table S1. Adjusted Day 22 postvaccination GMT, GMT ratio, seroconversion, and analysis of noninferiority of aIIV4 relative to IIV4 in subjects 50–64 years of age by HI Assay; Table S2. Day 22 adjusted postvaccination GMT, GMT ratio, and analysis of superiority in subjects 50–64 years of age by HI assay; Table S3. Unadjusted pre- and postvaccination GMT, GMFI, percentage of subjects with titer ≥ 1:40, and seroconversion rates in subjects 50–64 years of age by HI assay; Table S4. Day 181 and 271 adjusted postvaccination GMT and GMT ratio in subjects 50–64 years of age by HI assay.
