# Supplementary material for: Immunogenicity and Safety of MF59-Adjuvanted Quadrivalent Influenza Vaccine Compared with a Nonadjuvanted, Quadrivalent Influenza Vaccine in Adults 50–64 Years of Age
Source: Vaccines (Basel). 2023 Sep 26;11(10):1528. doi: 10.3390/vaccines11101528 (PMC10611124; doi:10.3390/vaccines11101528)
Supplement: Supplementary file 1 [file vaccines-11-01528-s001.zip › vaccines-2571129-supplementary.pdf]

Supplementary Materials for

Immunogenicity and Safety of MF59-adjuvanted Quadrivalent Influenza Vaccine Compared with a Nonadjuvanted, Quadrivalent Influenza Vaccine in Adults 50–64 Years of Age

Airi Poder, Janine Oberije, Jay Meyer, Peter Heymer, Deborah Molrine, Eve Versage, Leah Isakov, Qiuhong Zhang, and Matthew Hohenboken

Figure S1. Subject disposition

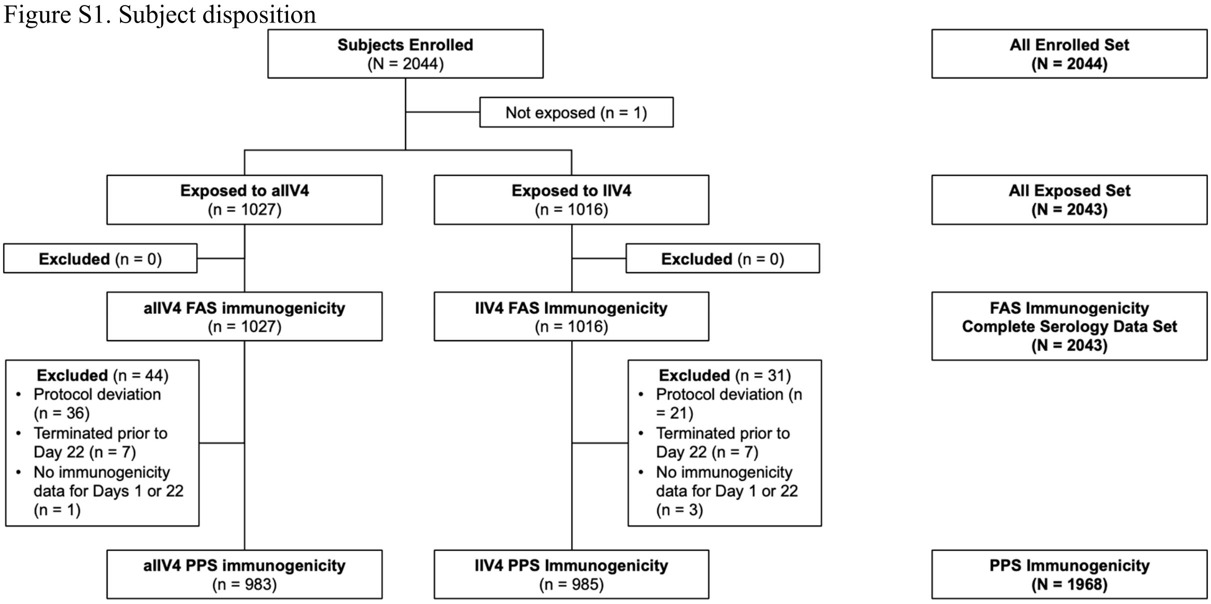

Table S1. Adjusted Day 22 postvaccination GMT, GMT ratio, seroconversion, and analysis of noninferiority of aIIV4 relative to IIV4 in subjects 50–64 years of age by HI Assay (PPS Immunogenicity).

	**Day 22 GMT (95% CI)**		**GMT ratio (IIV4/aIIV4) (95% CI)**
	**aIIV4 (n=983)**	**IIV4 (n=985)**	
A(H1N1)	731.90 (689.39–777.04)	586.85 (552.83–622.96)	0.802 (0.738–0.871)
A(H3N2)	347.89 (324.78–372.64)	313.16 (292.42–335.36)	0.900 (0.819–0.989)
B/Victoria	144.41 (136.97–152.26)	143.32 (135.97–151.07)	0.992 (0.923–1.067)
B/Yamagata	154.40 (146.80–162.40)	145.74 (138.57–153.27)	0.944 (0.880–1.012)
	**Day 22 SCR (95% CI)**		**SCR difference (IIV4 – aIIV4) (95% CI)**
	**aIIV4 (n=983)**	**IIV4 (n=985)**	
A(H1N1)	81.2 (78.57–83.58)	76.8 (74.04–79.42)	–4.4 (–7.97 to –0.74)
A(H3N2)	63.6 (60.46–66.63)	61.8 (58.61–64.82)	–1.8 (–6.14 to 2.48)
B/Victoria	44.5 (41.39–47.74)	40.6 (37.52–43.76)	–3.9 (–8.31 to 0.45)
B/Yamagata	43.4 (40.27–46.60)	41.0 (37.92–44.19)	–2.4 (–6.77 to 2.00)

Table S2. Day 22 adjusted postvaccination GMT, GMT ratio, and analysis of superiority in subjects 50–64 years of age by HI assay (FAS Immunogenicity).

	**Day 22 GMT (95% CI)**		**GMT ratio (IIV4/aIIV4) (95% CI)**
	**aIIV4 (n=983)**	**IIV4 (n=985)**	
A(H1N1)	729.17 (687.51–773.36)	589.07 (555.25–624.94)	0.808 (0.745–0.876)
A(H3N2)	347.09 (324.44–371.33)	315.69 (295.04–337.79)	0.910 (0.829–0.998)
B/Victoria	143.73 (136.44–151.42)	143.74 (136.42–151.45)	1.000 (0.931–1.075)
B/Yamagata	155.19 (147.67–163.09)	146.89 (139.74–154.40)	0.947 (0.884–1.014)

Table S3. Unadjusted pre- and postvaccination GMT, GMFI, percentage of subjects with titer ≥1:40, and seroconversion rates in subjects 50–64 years of age by HI assay (FAS Immunogenicity)

**Assessment**	**aIIV4 (n=1027)**	**IIV4 (n=1016)**
A(H1N1)		
Day 1 GMT, value (95% CI)	54.37 (49.71–59.47)	50.41 (46.13–55.09)
Day 22 GMT, value (95% CI)	708.36 (666.39–752.98)	553.70 (519.14–590.56)
Day 181 GMT, value (95% CI)	330.76 (308.44–354.70)	276.24 (257.32–296.56)
Day 22 GMFI, value (95% CI)	13.07 (11.92–14.34)	11.00 (10.07–12.02)
Day 181 GMFI, value (95% CI)	6.22 (5.71–6.76)	5.47 (5.03–5.95)
Day 1 HI titer ≥1:40, % (95% CI)	65.2 (62.20–68.15)	64.2 (61.14–67.15)
Day 22 HI titer ≥1:40, % (95% CI)	99.7 (99.14–99.94)	99.2 (98.44–99.66)
Day 181 HI titer ≥1:40, % (95% CI)	98.2 (97.13–98.92)	96.3 (94.89–97.36)
Day 22 SCR, % (95% CI)	80.8 (78.24–83.20)	77.1 (74.34–79.65)
Day 181 SCR, % (95% CI)	64.5 (61.46–67.55)	56.1 (52.94–59.24)
A(H3N2)		
Day 1 GMT, value (95% CI)	45.97 (42.06–50.25)	46.54 (42.63–50.81)
Day 22 GMT, value (95% CI)	323.40 (300.77–347.73)	292.50 (271.72–314.88)
Day 181 GMT, value (95% CI)	151.92 (141.27–163.37)	143.80 (133.33–155.09)
Day 22 GMFI, value (95% CI)	7.09 (6.43–7.83)	6.31 (5.78–6.89)
Day 181 GMFI, value (95% CI)	3.29 (3.04–3.56)	3.08 (2.86–3.31)
Day 1 HI titer ≥1:40, % (95% CI)	60.3 (57.16–63.30)	61.7 (58.61–64.72)
Day 22 HI titer ≥1:40, % (95% CI)	97.5 (96.39–98.40)	97.3 (96.12–98.23)
Day 181 HI titer ≥1:40, % (95% CI)	92.0 (90.08–93.59)	89.9 (87.81–91.68)
Day 22 SCR, % (95% CI)	63.4 (60.35–66.42)	61.8 (58.65–64.79)
Day 181 SCR, % (95% CI)	39.4 (36.28–42.54)	38.6 (35.51–41.72)
B/Victoria		
Day 1 GMT, value (95% CI)	36.24 (33.78–38.87)	37.06 (34.56–39.73)
Day 22 GMT, value (95% CI)	134.45 (126.24–143.21)	132.45 (124.32–141.10)
Day 181 GMT, value (95% CI)	74.17 (69.66–78.97)	75.99 (71.42–80.85)
Day 22 GMFI, value (95% CI)	3.73 (3.45–4.02)	3.60 (3.33–3.88)
Day 181 GMFI, value (95% CI)	2.06 (1.93–2.19)	2.05 (1.92–2.20)
Day 1 HI titer ≥1:40, % (95% CI)	58.5 (55.42–61.57)	60.4 (57.30–63.43)
Day 22 HI titer ≥1:40, % (95% CI)	94.4 (92.79–95.72)	93.3 (91.63–94.81)
Day 181 HI titer ≥1:40, % (95% CI)	83.4 (80.90–85.66)	84.3 (81.84–86.48)
Day 22 SCR, % (95% CI)	43.9 (40.86–47.08)	40.6 (37.50–43.68)
Day 181 SCR, % (95% CI)	24.3 (21.64–27.13)	23.6 (20.97–26.38)
B/Yamagata		
Day 1 GMT, value (95% CI)	38.50 (35.83–41.38)	38.33 (35.68–41.18)
Day 22 GMT, value (95% CI)	144.30 (136.02–153.08)	134.15 (126.31–142.48)
Day 181 GMT, value (95% CI)	76.87 (72.33–81.70)	77.34 (72.72–82.25)
Day 22 GMFI, value (95% CI)	3.77 (3.50–4.07)	3.50 (3.25–3.77)
Day 181 GMFI, value (95% CI)	2.03 (1.91–2.16)	2.01 (1.89–2.14)
Day 1 HI titer ≥1:40, % (95% CI)	60.8 (57.77–63.87)	61.9 (58.81–64.90)
Day 22 HI titer ≥1:40, % (95% CI)	95.9 (94.47–97.01)	94.6 (93.05–95.94)
Day 181 HI titer ≥1:40, % (95% CI)	83.9 (81.48–86.17)	84.0 (81.60–86.28)
Day 22 SCR, % (95% CI)	42.9 (39.82–46.02)	41.1 (37.99–44.19)
Day 181 SCR, % (95% CI)	22.4 (19.82–25.16)	22. 4 (19.86–25.19)

Table S4. Day 181 and 271 adjusted postvaccination GMT and GMT ratio in subjects 50–64 years of age by HI assay (FAS Immunogenicity)

	GMT (95% CI)		GMT ratio (IIV4/aIIV4) (95% CI)
	aIIV4 (n=1027)	IIV4 (n=1016)	
**Day 181**			
A(H1N1)	351.73 (331.61–373.06)	306.11 (288.62–324.66)	0.870 (0.803–0.944)
A(H3N2)	165.17 (155.85–175.05)	157.34 (148.48–166.73)	0.953 (0.880–1.032)
B/Victoria	79.51 (75.81–83.40)	81.73 (77.92–85.72)	1.028 (0.963–1.098)
B/Yamagata	83.42 (79.81–87.20)	84.53 (80.87–88.36)	1.013 (0.953–1.077)
**Day 271**			
A(H1N1)	188.37 (176.44–201.11)	168.05 (157.47–179.35)	0.892 (0.815–0.976)
A(H3N2)	100.50 (95.06–106.25)	95.01 (89.90–100.42)	0.945 (0.876–1.020)
B/Victoria	46.53 (44.66–48.49)	47.60 (45.69–49.60)	1.023 (0.967–1.083)
B/Yamagata	71.66 (69.20–74.20)	73.24 (70.74–75.83)	1.022 (0.974–1.072)
